# Shortening of the Lower Limb after Fracture of the Femur

**Published:** 1877-05

**Authors:** 


### Shortening of the Lower Limb after Fracture of the Femur.

Dr. Jarvis S. Wright, Professor of Surgery at the Long Island College Hospital, confirms (*Archives of Clinical Surgery*, Feb. 1877) the observations of Dr. C. Cox (*American Journal of the Medical Sciences*, April, 1875) as regards the inequality in length of the normal lower limbs in a large proportion of cases.

Dr. Wright's investigations have led him to the following conclusions:—

*First*, The lower limbs of the same persons are not always of the same length.

*Secondly*, The greater number of lower limbs, comparing the limbs of the same person, show a difference in length.

*Thirdly*, The normal limbs that I have measured give the following result: The left lower limb is oftener longer than the right lower limb, and the right lower limb is nearly as often longer than the left lower limb.

*Fourthly*, About one person out of every five has lower limbs that measure the same length.

*Fifthly*, The difference in the length of the normal lower limbs of the same person, varies in different cases—from one-eighth of an inch to one inch. I have considered every difference less than one-eighth of an inch as no difference. And I found one case in which the left limb measured one inch and three-eighths of an inch longer than the right limb; of course, I recognize the extreme difficulty of making exact measurements of lower limbs; but the same difficulty supervenes to perplex the measurements of lower limbs, one of which has a broken femur; and if it is proper and justifiable to make comparative measurements of broken bones, before and after union, it is admissible to make comparative measurements of unbroken bones. The facts are as reliable in one case as in the other.

*Sixthly*, I have measured the lower limbs of cadavers and of skeletons, the soft parts having been removed by dissection. These measurements confirm the above results.

If Dr. Wright's investigations and observations are correct—

I. To assume an equality in the normal length of the lower limbs of a patient who has a broken femur, would, in many cases, involve an error of more or less gravity. In other words, it would be quite impossible to say, from the most careful measurements, how much a broken femur had shortened a limb, if the uninjured limb is made the standard of comparison. And a correct and reliable slandard could only be reached in a case in which the injured limb had been measured *before* and *after* the injury. There are more chances against than for an assumption of an equality of length in the lower limbs of any one one case.

II. Suppose a case: A man's left lower limb measures one inch longer than his right lower limb, and he has never been injured. Let him fall and break his right femur. It would certainly be very unscientific, unskillful and perilous to apply extension and counter-extension so forcibly as to make the injured limb as long as the other. Having in mind the great strength and resistance of the facia of the thigh, I must say that in such a case such extension would be impossible; and, if it were possible, it would be unjustifiable. Or let him break his left femur. A moderate degree of extension and counter-extension would make the injured limb as long as the other, and readily keep it as long. Such cases as this are quite often met in one's surgical experience. Circumstances sometimes favor the surgeon, but they are quite as often against him. We get credit when we deserve it least, we get blame when we deserve most praise.

III. Practically, in some cases of fracture of the femur, I have easily made the injured limb as long as the other, and have supposed the good result to be partly due to skill and treatment. In other cases, I have found it absolutefy imposible, by any juitifiable means, to prevent as much as an inch shortening. Cases of extreme shortening after fracture of the femur have not fallen to my lot. A specimen of very great shortening of a broken femur, with firm union, you will find in the museum of the college. Now, I have often accused the muscles and fascia for interfering with my attempts to obtain perfect results. Why, the femur is playing the trick of evolution, it varies in length!

IV. I have always held, that in a certain number of cases of fracture of the femur, shortening will occur after any treatment, and have so testified before legal tribunals, usually putting the average shortening, when shortening takes place, at about one inch; and I must now add to the accidental and the ordinary causes of shortening in the cases of fracture of the femur, an important one, namely, a quite common inequality in the length of the lower limbs whose bones have never been broken.

V. If these results are based on sound observation, they effect materially suits at law for malpractice; for, if a patient has had a fracture of the femur, and the corresponding limb be an inch or more shorter than the other after treatment; and if the patient feels himself aggrieved and sues his surgeon, on account of the apparently unusual shortening; and if it can be shown, that in a considerable number of cases, lower limbs whose femora have not been broken are not of equal length, then the surgeon has a good cause; in fact, he is unquestionably armed with a strong defence over and above his having used all due dilligence in the care of his patient. Because, no surgeon can be expected to repair a broken bone so that it will be more perfect than it was originally. And I need not tell you that these facts bear heavily on any one who may claim the skill and the ability to prevent shortening, in the treatment of all cases of fracture of the femur; and who may boast of having so applied the art of setting broken bones, that he has actually made the broken bone longer than the corresponding unbroken one. In fact, I have treated two cases of fracture of the femur, occurring within two days of each other; the patients were put in beds side by side; one was a boy, the other was an adult; the adult had the minimum of surgical attention, and recovered with lower limbs of the same length; the boy had the maximum of surgical attention, and after union of the fragments of bone, the injured limb was one inch shorter that the other. Let me add, that the extending weight for the boy was half as heavy again as that for the adult.

Dr. Wright thinks we are entitled to make the following practical conclusions:—

1. We need not expect in all cases of fracture of the femur to give the patients lower limbs of equal length. In other words we cannot always prevent the so-called *shortening*; the number of shortened limbs cannot be accurately fixed.

2. In a certain number of cases of fracture of the femur the injured limb will remain shorter than the other—no matter what the treatment may have been.

2. Excessive efforts persisted in to bring the injured limb down and make it as long as the uninjured one will sometimes fail, and are calculated to do harm; since the strong fascia of the thigh offers great resistance, and since the injured limb may have been shorter than the other before injury.

4. If need be, complete relaxation of the powerful muscles of the thigh by etherization will enable an ordinary and admissible degree of extension and counter-extension to give the injured limb a maximum length; or extending weights gradually applied will "tire out" the muscles; at first apply four pounds, then add to that four more pounds, then make the weight twelve pounds, now increase the extension to sixteen pounds, and in some instances make the extending weight twenty pounds, removing a certain part of the extension as may be considered necessary.

5. The possibility of having the injured limb longer after treatment than the other must be recognized, and the most probable explanation of such a result must be given.

6. These conclusions conform to the practice and agree with the results of the best surgeons.

Finally, perhaps I ought to add, that the variation in the length and obliquity of the neck of the femur, incident to the age of the patient may not occur during the same time and with equal pace in the femoral necks, and that this may be one cause in some instances of a difference in the normal length of lower limbs. At any rate it may be noted that there is a remarkable approach to an agreement between tne differences in the length of normal lower limbs, and the difference in length of lower limbs—one of which has had the femur broken ; only the average difference is somewhat greater in case there has been a fracture of the femur. But, in general, the tendency of a fracture of the femur is to shorten the limb to which it belongs. And we may fairly regard assertions of always having lower limbs of equal length, after treating fracture of the femur, as open to just criticism. Such assertions are calculated to put individual surgeons in peril of suits at law for malpractice when they do not deserve it; and they are, if found to be untrue, a sure means of throwing discredit on a useful and honorable profession.—*Monthly Abstract Medical Science.*

